# Plasma lipidome, circulating inflammatory proteins, and Parkinson’s disease: a Mendelian randomization study

**DOI:** 10.3389/fnagi.2024.1424056

**Published:** 2024-09-11

**Authors:** Yidan Qin, Lin Wang, Jia Song, Wei Quan, Jing Xu, Jiajun Chen

**Affiliations:** Department of Neurology, China-Japan Union Hospital of Jilin University, Changchun, Jilin, China

**Keywords:** Parkinson’s disease, plasma lipidome, circulating inflammatory proteins, Mendelian randomization, causal relationship

## Abstract

**Background:**

Observational studies have suggested that plasma lipidome play a pivotal role in the occurrence of Parkinson’s disease (PD). However, it remains unknown which lipids among plasma lipidome affect PD and how they exert their influence. Clarity is lacking regarding the causal relationship between plasma lipidome and PD, as well as whether circulating inflammatory proteins serve as mediators.

**Methods:**

Single nucleotide polymorphisms (SNPs) significantly associated with 179 plasma lipidome were selected as instrumental variables to assess their causal impact on PD. PD data, serving as the outcome, were sourced from the International Parkinson’s Disease Genomics Consortium, which boasts the largest sample size to date. The inverse variance weighted (IVW), Weighted median method, MR-Egger method, Simple mode method, Weighted mode method and MR-PRESSO were employed to evaluate the influence of the 179 plasma lipidome on PD. Heterogeneity, pleiotropy tests, and reverse causality analyses were conducted accordingly. Additionally, we analyzed the causal relationship between 91 circulating inflammatory proteins and PD, exploring whether these proteins serve as mediators in the pathway from plasma lipidome to PD.

**Results:**

Among the 179 plasma lipidome, three were found to be associated with a reduced risk of PD: Phosphatidylcholine (14:0_18:2) (IVW, OR = 0.877; 95%CI, 0.787–0.978; *p* = 0.018), Phosphatidylcholine (16:0_16:1) levels (IVW, OR = 0.835; 95%CI, 0.717–0.973; *p* = 0.021), and Phosphatidylcholine (O-17:0_17:1) levels (IVW, OR = 0.854; 95%CI, 0.779–0.936; *p* = 0.001). Meanwhile, Sphingomyelin (d38:1) was linked to an increased risk of PD (IVW, OR = 1.095; 95%CI, 1.027–1.166; *p* = 0.005). Among the 91 circulating inflammatory proteins, three were associated with a lower PD risk: Fibroblast growth factor 21 levels (IVW, OR = 0.817; 95%CI, 0.674–0.990; *p* = 0.039), Transforming growth factor-alpha levels (IVW, OR = 0.825; 95%CI, 0.683–0.998; *p* = 0.048), and Tumor necrosis factor receptor superfamily member 9 levels (IVW, OR = 0.846; 95%CI, 0.744–0.963; *p* = 0.011). Two were associated with a higher risk of PD: Interleukin-17A levels (IVW, OR = 1.285; 95%CI, 1.051–1.571; *p* = 0.014) and TNF-beta levels (IVW, OR = 1.088; 95%CI, 1.010–1.171; *p* = 0.026). Additionally, a positive correlation was observed between Phosphatidylcholine (14:0_18:2) levels and Fibroblast growth factor 21 levels (IVW, OR = 1.125; 95%CI, 1.006–1.257; *p* = 0.038), suggesting that Fibroblast growth factor 21 levels may serve as a mediating factor in the pathway between Phosphatidylcholine (14.0_18.2) levels and PD. The mediation effect was estimated to be −0.024, accounting for approximately 18% of the total effect.

**Conclusion:**

Both plasma lipidome and circulating inflammatory proteins demonstrate a causal relationship with PD. Additionally, circulating inflammatory proteins may serve as mediators in the pathway from plasma lipidome to PD. These findings may contribute to the prediction and diagnosis of PD and potentially pave the way for targeted therapies in the future.

## 1 Introduction

Parkinson’s disease (PD) is a progressive neurodegenerative disorder characterized by the loss of dopaminergic neurons in the substantia nigra and the deposition of α-synuclein. Its typical manifestations include resting tremor, bradykinesia, postural instability, and rigidity of the limbs (Tansey et al., 2022). With the aging population, the prevalence of PD is expected to gradually increase (Beitz, 2014), potentially doubling over the next 30 years (Tolosa et al., 2021), posing a significant burden on patients’ daily activities and society’s healthcare system. However, the pathogenesis of PD remains unclear, early diagnosis is challenging, and current treatments are not curative. Therefore, it is crucial to explore predictive factors for PD to improve early diagnosis rates and develop better targeted therapies.

Lipids, an essential component of cell membranes, are primarily classified in mammals as glycerides, sphingolipids, and sterols. Initially regarded as structural components, lipids have been found to regulate various cellular physiological functions and play a crucial role in cell signaling and the production of bioactive metabolites (Cockcroft, 2021). Studies have shown that lipid metabolism is not only associated with immune diseases, cardiovascular diseases, and diabetes (Luo et al., 2008; Duan et al., 2022; Zhang et al., 2022), but also intimately linked to PD. Decreased catabolism of lipid substrates in lysosomes can affect lysosomal function, thereby impeding the clearance of α-synuclein (Galper et al., 2022). Additionally, lipids can mediate the onset of PD by regulating immune responses, oxidative stress, endolysosomal function, and endoplasmic reticulum stress (Xicoy et al., 2019). Notably, lipids are part of a complex network regulating numerous cellular and molecular processes, particularly inflammation, playing a significant role in modulating inflammatory factors (Gonçalves et al., 2012; Leuti et al., 2020).

Neuroinflammation is a key pathogenic mechanism in PD (Minchev et al., 2022; Morris et al., 2024). Numerous studies have demonstrated that inflammatory factors can modulate the occurrence of neuroinflammation, thus participating in the pathogenesis of PD (Wang et al., 2019; Gautam et al., 2023). Both neurohistological and neuroimaging studies support the persistent presence of neuroinflammatory processes throughout the development and terminal stages of PD. Inflammatory markers in peripheral blood and cerebrospinal fluid may also trigger or exacerbate neuroinflammation, leading to neurodegeneration (Tansey et al., 2022). Furthermore, Belarbi et al. (2020) suggested that metabolic dysregulation of glycosphingolipids may be associated with the occurrence of neuroinflammation in PD.

Research indicates altered plasma lipidome profiles in PD patients (Guo et al., 2015), suggesting a possible association between PD and plasma lipidome metabolism disorders (Hu et al., 2020). Although several meta-analyses have shown that serum triglyceride, low-density lipoprotein cholesterol, and total cholesterol levels have a protective effect on PD (Fu et al., 2020; Jiang et al., 2020; Lu et al., 2021; Hong et al., 2022), these are based solely on observational studies, and the causal relationship between these lipids and PD remains unclear. It is also unknown which other lipids are associated with PD and the underlying mechanisms involved. Additionally, meta-analyses have demonstrated significantly elevated IL-17 levels in PD patients (Gautam et al., 2023), leading us to speculate that circulating inflammatory proteins may mediate the pathway from plasma lipidome to PD. The identification of plasma lipidome may aid in the prediction and diagnosis of PD and potentially serve as therapeutic targets for PD in the future. Therefore, we conducted a Mendelian randomization study to address these questions.

Mendelian randomization (MR) is a genetic analytical approach that relies on the random allocation of parental alleles to offspring. It aims to estimate the causal relationship between a specific exposure and outcome by using genetic variations in exposure as instrumental variables. Serving as a natural randomized controlled trial (RCT), MR can reduce the confounding effects of environmental factors and reverse causality inherent in observational studies (Skrivankova et al., 2021). In this study, we conducted a comprehensive MR analysis using the latest and largest genome-wide association studies (GWAS) for 179 plasma lipidome, 91 circulating inflammatory proteins, and PD. This analysis aimed to reveal the causal relationship between these plasma lipidome and PD risk and explore whether the circulating inflammatory proteins serve as mediators in the pathway from plasma lipidome to PD.

## 2 Materials and methods

This study is a re-analysis of previously collected and published data and does not require additional ethical approval.

### 2.1 Study design

**Figure 1:**
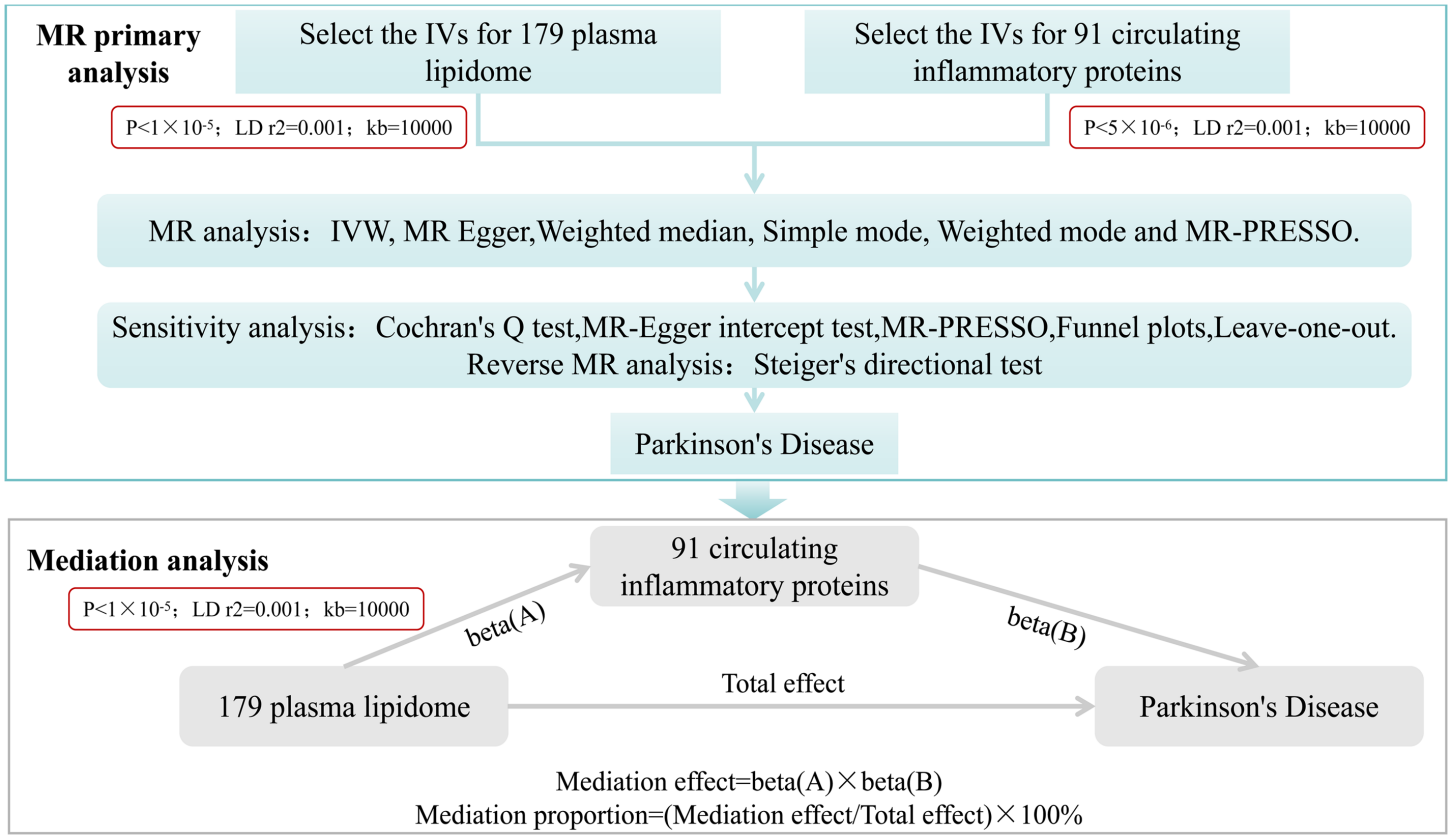
Study desige. Overview of the research design. It is mainly divided into two parts: MR primary analysis and mediation analysis.

This study mainly comprises two parts. Firstly, we analyzed the causal effects of 179 plasma lipidome groups and 91 circulating inflammatory proteins on PD, respectively, and conducted sensitivity analysis and reverse analysis. Secondly, we employed a two-step and multivariate MR (MVMR) analysis to investigate the mediating role of circulating inflammatory proteins in the relationship between plasma lipidome and PD (Figure 1).

### 2.2 Data source

The genetic data for plasma lipidome were derived from a 2023 GWAS summary dataset encompassing 7,174 Finnish individuals. This comprehensive dataset includes 179 lipid species belonging to 13 lipid classes covering 4 major lipid categories: glycerolipids, glycerophospholipids, sphingolipids, and sterols (Ottensmann et al., 2023). On the other hand, the genetic data for circulating inflammatory proteins were sourced from a 2023 GWAS study involving 14,824 European individuals across 11 cohorts. This dataset encompasses 91 inflammation-related plasma proteins (Zhao et al., 2023).

The GWAS summary data for PD were obtained from the International Parkinson’s Disease Genomics Consortium, which has been archived by the ieu open gwas project (GWAS ID: ieu-b-7). Representing the largest sample size available thus far, this dataset comprises 33,674 PD patients and 449,056 control cases, encompassing 14 cohorts (Nalls et al., 2019).

### 2.3 Instrumental variables selection

In MR analysis, the instrumental variables (IVs) selected must satisfy three assumptions: first, the genetic variant must be strongly associated with the exposure; second, it must be independent of confounding factors; and third, its effect on the outcome can only be mediated through the exposure (Skrivankova et al., 2021).

Using the R package TwoSampleMR (version 0.5.7), SNPs significantly associated with plasma lipidome were chosen with a threshold of *p* < 1 × 10^−5^, while SNPs significantly associated with circulating inflammatory proteins were selected using a threshold of *p* < 5 × 10^−6^. Subsequently, SNPs in linkage disequilibrium were excluded based on the criteria of r2 < 0.001 and a distance of >10,000 kb. After matching, palindromic SNPs were further removed. The resulting selected IVs were then utilized for MR estimation of the causal relationships between plasma lipidome or circulating inflammatory proteins and PD.

Additionally, we calculated the F-statistic for each IV and discarded weak IVs with an F-statistic <10 (Burgess et al., 2017).

### 2.4 MR primary analysis

To evaluate the causal impacts of plasma lipidome and circulating inflammatory proteins on PD, we conducted two-sample Mendelian randomization (MR) analyses using R version 4.3.1 and the TwoSampleMR package (version 0.5.7) for plasma lipidome and circulating inflammatory proteins, respectively. Inverse-variance weighted (IVW) analysis was employed as the primary analytical approach. Additionally, we complemented our findings using MR Egger, weighted median, simple mode, weighted mode and MR-PRESSO. Heterogeneity among the selected IVs was tested using the Cochran’s Q test. Multicollinearity was examined using the MR-Egger test, with a significant intercept term indicating horizontal pleiotropy (Burgess and Thompson, 2017). Furthermore, we employed the Mendelian randomization pleiotropy residual sum and outlier (MR-PRESSO) approach to assess pleiotropy and exclude outliers that could potentially bias our estimates (Verbanck et al., 2018). Finally, scatter plots, funnel plots, and leave-one-out sensitivity tests were generated to further validate our results. Statistical significance was considered when the *p*-value from IVW was less than 0.05 and the directions of IVW and MR Egger were concordant. These rigorous analytical approaches ensure the robustness and reliability of our findings.

### 2.5 Bi-directional causality analysis

To assess the impact of reverse causality on our findings, we performed MR analysis with PD as the exposure and plasma lipidome or circulating inflammatory proteins associated with PD as the outcomes. SNPs significantly associated with PD were selected using a threshold of *p* < 1 × 10^−5^, with the remaining steps identical to the forward analysis. Furthermore, the Steiger’s directional test was also conducted to further verify the correctness of the causal relationship direction.

### 2.6 Mediation analysis

After completing MR primary analysis, we incorporated those plasma lipidome and circulating inflammatory proteins that exhibited significant causal effects on PD without any reverse causality into step 2. we employed a two-step MR approach and further examined the causal relationships between PD-associated plasma lipidome and PD-related circulating inflammatory proteins. The threshold for selecting SNPs significantly associated with plasma lipidome was set at *p* < 1 × 10^−5^. Similarly, results were considered statistically significant when the *p*-value from IVW analysis was less than 0.05 and the directions of IVW and MR Egger were concordant. Under these conditions, the mediation effect was calculated as Beta(A) * Beta(B), and the proportion of mediation effect was expressed as a percentage of the total effect (Figure 1). Finally, a multivariate Mendelian randomization analysis was performed.

Additionally, we conducted a reverse causality analysis by treating the circulating inflammatory proteins as the exposure and plasma lipidome as the outcome, using the same MR analytical approach as in the forward analysis.

## 3 Results

### 3.1 The causal effects of 179 plasma lipidome on PD

**Figure 2:**
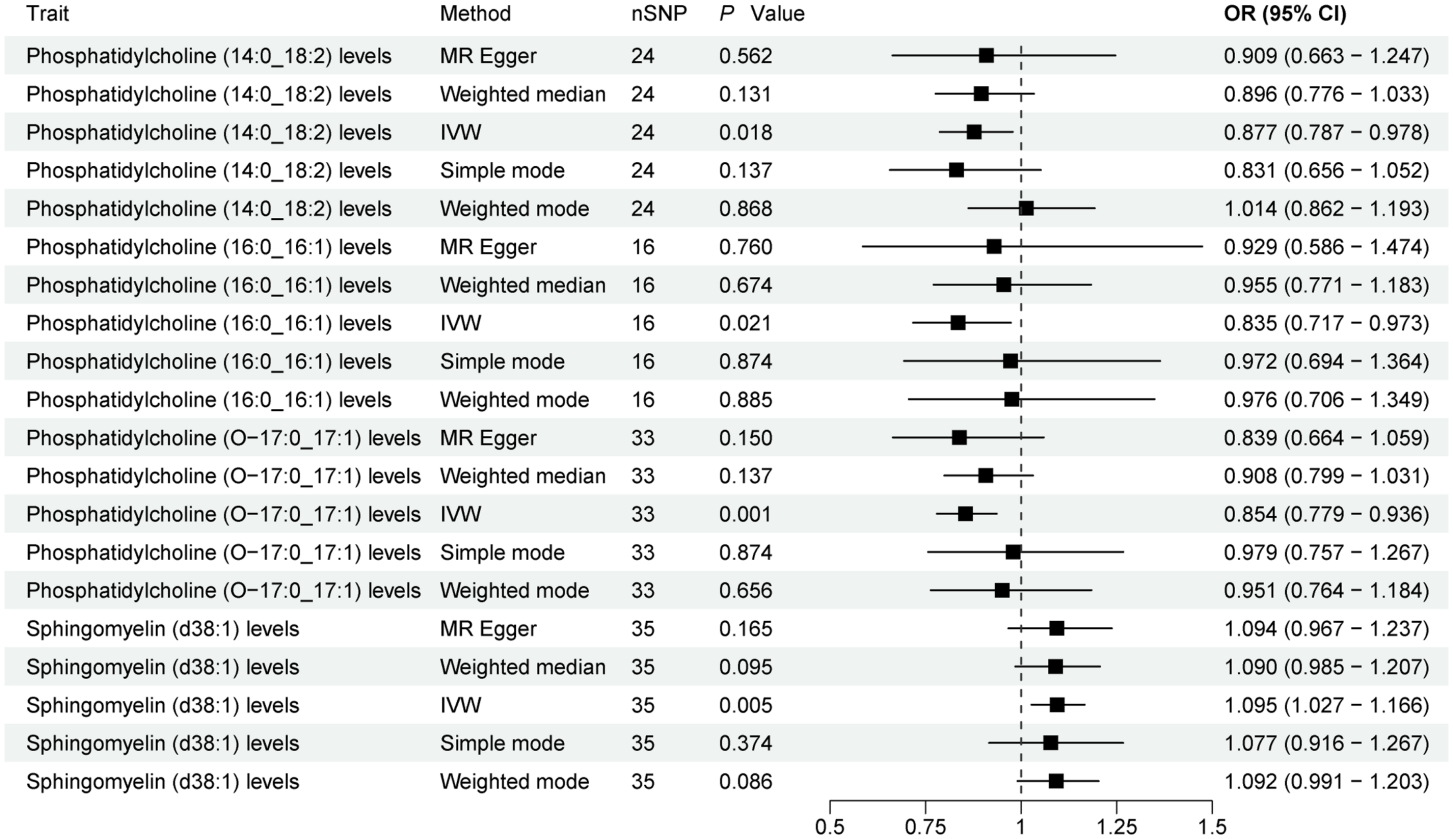
Forest plot of 179 plasma lipidome and PD. Forest plots showed the causal associations between plasma lipidome and PD by using different methods. IVW, inverse variance weighting; CI, confidence interval; OR, odds ratio.

The present study comprehensively analyzed the association between 179 plasma lipidome and PD, with 3,981 SNPs being selected as instrumental variables (IVs) for the 179 plasma lipidome (Supplementary Table S1). The results (Figure 2; Supplementary Table S2) revealed that three plasma lipidome were significantly associated with a reduced risk of PD. Notably, these three plasma lipidome belonged to different subtypes within the same category. Specifically, (1) higher levels of Phosphatidylcholine (14:0_18:2) exhibited a negative correlation with PD risk (IVW, OR = 0.877; 95%CI, 0.787–0.978; *p* = 0.018). (2) Similarly, increased Phosphatidylcholine (16:0_16:1) levels were inversely associated with PD risk (IVW, OR = 0.835; 95%CI, 0.717–0.973; *p* = 0.021). (3) Furthermore, Phosphatidylcholine (O-17:0_17:1) levels also demonstrated a negative association with PD risk (IVW, OR = 0.854; 95%CI, 0.779–0.936; *p* = 0.001).

Contrarily, one plasma lipidome was identified to be positively associated with an increased risk of PD (Figure 2). Specifically, higher levels of Sphingomyelin (d38:1) were positively correlated with PD risk (IVW, OR = 1.095; 95%CI, 1.027–1.166; *p* = 0.005). Finally, the results were further verified using MR-PRESSO, and all results showed *p* < 0.05 (Supplementary Table S3).

### 3.2 The causal effects of 91 circulating inflammatory proteins on PD

**Figure 3:**
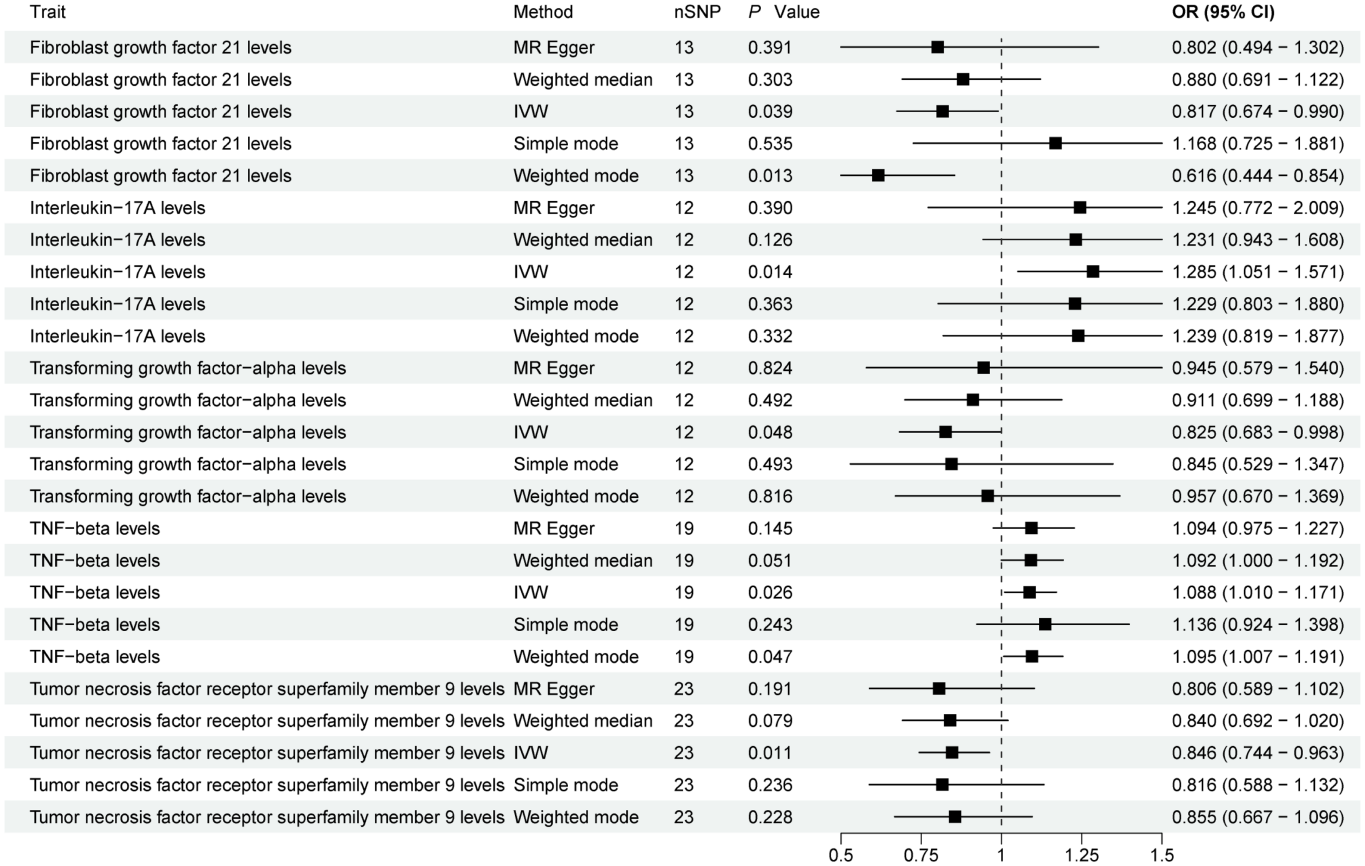
Forest plot of 91 circulating inflammatory proteins and PD. Forest plots showed the causal associations between circulating inflammatory proteins and PD by using different methods. IVW, inverse variance weighting; CI, confidence interval; OR, odds ratio.

The present study comprehensively examined the association between 91 circulating inflammatory proteins and PD, with 1,435 SNPs selected as instrumental variables (IVs) for the 91 circulating inflammatory proteins (Supplementary Table S4). The results (Figure 3; Supplementary Table S5) revealed that three circulating inflammatory proteins were significantly associated with a reduced risk of PD. Specifically, (1) higher levels of Fibroblast growth factor 21 levels exhibited a negative correlation with PD risk (IVW, OR = 0.817; 95%CI, 0.674–0.990; *p* = 0.039). (2) Similarly, increased levels of Transforming growth factor-alpha levels were inversely associated with PD risk (IVW, OR = 0.825; 95%CI, 0.683–0.998; *p* = 0.048). (3) Furthermore, Tumor necrosis factor receptor superfamily member 9 levels also demonstrated a negative association with PD risk (IVW, OR = 0.846; 95%CI, 0.744–0.963; *p* = 0.011).

Contrarily, two circulating inflammatory proteins were identified to be positively associated with an increased risk of PD (Figure 3). Specifically, (1) higher levels of Interleukin-17A levels were positively correlated with PD risk (IVW, OR = 1.285; 95%CI, 1.051–1.571; *p* = 0.014). (2) Additionally, TNF-beta levels also exhibited a positive association with PD risk (IVW, OR = 1.088; 95%CI, 1.010–1.171; *p* = 0.026). Finally, the results were further verified using MR-PRESSO (Supplementary Table S3).

### 3.3 Sensitivity analyses and reverse causal effects of PD on plasma lipidome and circulating inflammatory proteins

In the Cochran’s Q test, both MR-Egger and IVW yielded *p*-values greater than 0.05, indicating no heterogeneity. Furthermore, the MR-Egger intercept test showed a *p*-value above 0.05, and MR-PRESSO reported a global *p*-value also exceeding 0.05, collectively suggesting the absence of pleiotropy (Supplementary Table S6). No significant abnormalities were observed in the leave-one-out analysis, forest plots, Scatter plots, and funnel plots (Supplementary Figures S1–S8).

Additionally, a reverse causality analysis was conducted with PD as the exposure and four lipid profiles and five circulating inflammatory proteins as the outcomes. The results demonstrated no causal relationship between PD and these analytes (Supplementary Table S7).

Finally, the Steiger’s directional test was conducted to further validate the correctness of the causal relationship directions between plasma lipidome, circulating inflammatory proteins and PD, respectively. The results showed that snp_r2.exposure was greater than snp_r2.outcome, indicating a stronger association between SNPs and exposure variables. All correct_causal_direction values were TRUE, and *p* < 0.05, indicating that the inferred causal directions were correct (Supplementary Table S8).

### 3.4 Mediation analysis

A two-step MR analysis was conducted to explore the mediating effects of plasma lipidome on PD via circulating inflammatory proteins. The results (Supplementary Table S9), revealed a positive correlation between Phosphatidylcholine (14:0_18:2) levels and Fibroblast growth factor 21 levels (IVW, OR = 1.125; 95%CI, 1.006–1.257; *p* = 0.038). Notably, both Phosphatidylcholine (14:0_18:2) levels and Fibroblast growth factor 21 levels exhibited a negative association with the risk of PD. Based on these findings, this suggests that Fibroblast growth factor 21 levels may serve as a mediator in the pathway linking Phosphatidylcholine (14:0_18:2) levels and PD, with a mediation effect of −0.024 and total effect of −0.131, the mediation effect accounts for approximately 18% of the total effect.

Additionally, when the circulating inflammatory proteins were considered as the exposure and the plasma lipidome as the outcome, MR analysis did not reveal any reverse causal influence (IVW, *p* = 0.116). Moreover, the results of the Steiger’s directional test showed that snp_r2.exposure was 0.103, snp_r2.outcome was 0.009, correct_causal_direction was TRUE, and *p* value was 3.96E-57, indicating that the inferred causal direction was correct.

Finally, a multivariate MR analysis was performed with Phosphatidylcholine (14:0_18:2) levels and Fibroblast growth factor 21 levels as exposures and PD as the outcome. Unfortunately, both exposure results were negative (*p* = 0.889 and *p* = 0.395).

## 4 Discussion

The brain ranks second only to adipose tissue in terms of lipid concentration and diversity. Within the central nervous system, metabolic disturbances of lipids have been linked to the occurrence, progression, and severity of PD (Castellanos et al., 2021). Furthermore, research has identified a shared genetic risk between lipids, lipoproteins, and PD, where genetic variations associated with PD regulate the blood levels of specific lipid species that play crucial roles in the pathogenesis of PD (Klemann et al., 2017; Xicoy et al., 2021).

Investigations have shown that abnormal metabolism of lipids such as triglycerides, glycerophosphoethanolamine, diglycerides, polyunsaturated fatty acids, sphingolipids, gangliosides, glycerophospholipids, and cholesterol can lead to aberrant formation of α-synuclein, triggering neuroinflammation in PD through various innate and adaptive immune responses (Hatton and Pandey, 2022). Notably, glycerophosphoethanolamine has been found to facilitate the binding of acidic phospholipids with α-synuclein, thereby promoting its abnormal aggregation (Jo et al., 2000). Sphingolipids, a specialized class of lipids primarily confined to the nervous system, play a pivotal role in inflammatory diseases, including PD (Quinville et al., 2021). Ceramide, a central molecule in sphingolipid metabolism, serves as a crucial regulator of cellular functions and can be degraded into sphingosine (Ventura et al., 2019). Research has demonstrated that the synthesis of ceramide, sphingosine, and sphingosine-1-phosphate is associated with α-synuclein aggregation in PD patients (Gaspar et al., 2018). Low levels of ceramide have also been associated with α-synuclein aggregation in PD (Abbott et al., 2014), suggesting that the conversion of ceramide to sphingosine may be a crucial step in α-synuclein aggregation in PD (Hatton and Pandey, 2022).

Scholars such as Avisar et al. (2021) have employed machine learning algorithms to analyze 517 lipids across 37 categories and discovered that dihydrosphingomyelin (dhSM-20:0), plasmalogen phosphatidylethanolamine (PEp-38:6; 42:7), glucosylceramide (GlcCer-16:0; 24:1), dihydroglycosphingosine-based ceramide (dhGB3-22:0;16:0), and to a lesser extent, dihydroganglioside GM3 (dhGM3-16:0), can aid in predicting the severity of PD. Additionally, Avisar et al. (2021) have demonstrated that various lipids, such as those derived from vegetable oils, animal fats, or fatty acids, can suppress cytotoxicity induced by pathological processes including mitochondrial dysfunction, oxidative stress, apoptosis, and inflammation. Targeted use of these oils or fatty acids may thus contribute to the prevention of neurodegenerative diseases. Furthermore, Alarcon-Gil et al. (2022) have shown that linoleic acid exhibits neuroprotective and anti-inflammatory effects in PD models. Exploring the relationship between lipids and PD not only facilitates the identification of predictive factors for early diagnosis and treatment but also aids in discovering therapeutic targets for PD. However, the intricate interplay between PD and lipid metabolism precludes a straightforward analysis of the plasma lipidome that influence PD through observational studies. As a result, the exact causal relationships and underlying mechanisms remain elusive. To address this, we employed a two-sample MR analysis to investigate the causal relationship between 179 plasma lipidome and PD, identifying four plasma lipidome with significant causal associations. Reverse causation analysis was also conducted. Nevertheless, the precise mechanisms underlying how these plasma lipidome contribute to PD remain unknown. We hypothesize that circulating inflammatory proteins may mediate the relationship between these plasma lipidome and PD. Consequently, we further analyzed the causal relationships between 91 circulating inflammatory proteins and PD, discovering five circulating inflammatory proteins with significant causal associations, which were also subjected to reverse causation analysis. Moreover, our MR analysis revealed a causal link between PD-associated plasma lipidome and PD-related circulating inflammatory proteins, suggesting that these circulating inflammatory proteins may serve as mediators in the pathway between plasma lipidome and PD. However, further multivariate MR analysis showed no correlation between the phosphatidylcholine (14:0_18:2), Fibroblast growth factor 21 and PD, and in the future, we still need more data to verify this result.

The MR results indicated that elevated serum levels of phosphatidylcholine (14:0_18:2), phosphatidylcholine (16:0_16:1), and phosphatidylcholine (O-17:0_17:1) were associated with a reduced risk of PD. Phosphatidylcholine, a zwitterionic phospholipid composed of a hydrophilic head and hydrophobic tail, is a crucial component of eukaryotic cell membranes. Its anti-inflammatory properties have been implicated in the treatment of ulcerative colitis (Treede et al., 2007). Additionally, phosphatidylcholine plays a pivotal role in intracellular cholesterol transport and maintaining membrane lipid homeostasis (Lagace, 2015). Genetic studies have shown a shared etiology and significant negative correlation between PD and blood levels of phosphatidylcholine aa 32:3 (Xicoy et al., 2021). Farmer et al. (2015) observed downregulation of phosphatidylcholine and lysophosphatidylcholine lipid classes in the substantia nigra of a PD animal model. Consistently, Chang et al. (2022) reported a downregulation of phosphatidylcholine (35:6) in the plasma of PD patients. However, López de Frutos et al. (2022) found increased levels of phosphatidylcholine and decreased levels of lysophosphatidylcholine in the plasma of PD patients from the Iberian Peninsula, which may be attributed to the species diversity of phosphatidylcholine and its varying roles in different metabolic pathways.

Furthermore, through a more profound analysis, it has been revealed that Fibroblast growth factor 21 levels may serve as a mediating factor in the pathway between phosphatidylcholine (14:0_18:2) and PD. Elevated levels of phosphatidylcholine (14:0_18:2) promote the increase of Fibroblast growth factor 21 levels, thereby exerting a protective effect on PD. The mediating effect is estimated to be −0.024, accounting for approximately 18% of the total effect. Research has shown that the phospholipase/lysophospholipase PNPLA8-PNPLA7 axis can break down phosphatidylcholine, enabling the endogenous choline stored in hepatic phosphatidylcholine to be utilized for methyl metabolism and providing methyl groups for the methionine cycle. In PNPLA7-deficient mice, due to reduced phosphatidylcholine breakdown, methionine deficiency and disrupted methionine cycling trigger the expression of Fibroblast growth factor 21 levels, mediating metabolic alterations (Hirabayashi et al., 2023). Fibroblast growth factor 21 levels have been proven to ameliorate brain metabolic disorders and behavioral deficits in PD mouse models by promoting a favorable colonic microbiota composition and influencing the microbiota-gut-brain metabolic axis (Yang et al., 2023). Additionally, it regulates microglial polarization through the sirtuin 1 (SIRT1)/nuclear factor-κ B (NF-κB) pathway, thus alleviating neurodegeneration in PD mice models and PD cellular models (Yang et al., 2021). Therefore, We hypothesize that an increase in phosphatidylcholine (14:0_18:2) levels may lead to a reduction in the availability of methyl groups for the methionine cycle, impeding the regeneration of methionine and the methyl donor S-adenosylmethionine. This disruption in the methionine cycle, in turn, promotes an increase in Fibroblast growth factor 21 levels, exerting a protective effect against PD. Therefore, a decrease in phosphatidylcholine (14:0_18:2) levels may serve as a predictor for PD.

Our MR analysis revealed that the risk of PD increases with elevated serum levels of sphingomyelin (d38:1). Sphingomyelin, a sphingolipid composed of ceramide linked to phosphocholine (or phosphoethanolamine) at the C-1 hydroxyl group, is synthesized from palmitic acid and serine through the intermediate sphingosine, followed by conjugation with acyl-CoA and phosphocholine. Prior studies have demonstrated a shared genetic etiology and positive correlation between PD and blood levels of Sphingomyelin26:0 (Xicoy et al., 2021). Consistent with our findings, Gusev et al. (2020) observed increased concentrations of seven out of 12 sphingomyelins in the blood of preclinical subjects with PD risk. Xicoy et al. (2020a) also reported increased levels of three sphingomyelin species in a PD cell model. However, contrasting results have emerged from lipid and transcriptome analyses of putamen samples from PD patients, revealing decreased levels of most sphingomyelins, particularly saturated sphingomyelins (Xicoy et al., 2020b). Similarly, Chang et al. (2022) analyzed lipid changes in the plasma of PD patients and found downregulation of sphingomyelins (d30:1), (d32:1), and (d39:1). The differences in these results may be related to the species diversity of sphingomyelins.

Sphingomyelins play diverse roles in PD, yet their specific functions remain elusive due to the complexity of their metabolic pathways. Decreased levels of acid sphingomyelinase in PD may lead to sphingomyelin accumulation, causing cellular toxicity. In addition, reduced neutral sphingomyelinase activity may be associated with decreased exocytosis of α-synuclein-containing exosomes, leading to intracellular accumulation of α-synuclein (Signorelli et al., 2021). Mutations in the sphingomyelin phosphodiesterase (SMPD1) gene, which cleaves the phosphocholine head group of sphingomyelin to produce ceramide (Schuchman et al., 1992), have been identified as a risk factor for PD (Foo et al., 2013; Mao et al., 2017). Low ceramide levels are also associated with α-synuclein aggregation in PD (Abbott et al., 2014). These suggesting that SMPD1 mutations may disrupt the conversion of sphingomyelin to ceramide, leading to sphingomyelin accumulation, ceramide depletion, and subsequent α-synuclein aggregation, which may trigger neuroinflammation and the occurrence of PD.

We further explored the mediating role of circulating inflammatory proteins between sphingomyelin (d38:1) and PD, but unfortunately, no positive conclusions were obtained. The relationship between sphingomyelin (d38:1) and PD may involve an exceptionally complex metabolic network, necessitating further investigation.

To the best of our knowledge, this study is the first to utilize MR analysis to investigate the causal relationship between 179 plasma lipidome and PD, and it was discovered that circulating inflammatory proteins may serve as intermediates. Our findings are reliable, grounded in MR analysis of published large-scale GWAS results, which circumvents issues of reverse causality and confounding factors. We identified four plasma lipidome with a causal link to PD and demonstrated that phosphatidylcholine (14:0_18:2) levels may reduce PD risk by promoting fibroblast growth factor 21 levels. This discovery may lead to the development of predictive biomarkers for PD, enhancing early diagnosis rates and facilitating the development of targeted therapeutics. However, several limitations should be acknowledged. Firstly, as the GWAS data used in this study were derived from European populations, our findings may not be generalizable to other ethnic groups. Secondly, the 179 plasma lipidome and 91 circulating inflammatory proteins analyzed were measured in blood, not cerebrospinal fluid. Future studies should expand sample sizes, include cerebrospinal fluid analysis, and investigate diverse populations to further elucidate the mechanisms and pathways underlying the role of plasma lipidome in PD. Finally, our mediation analysis revealed that further multivariate MR analysis showed no correlation between phosphatidylcholine (14:0_18:2), fibroblast growth factor 21, and PD. More data are still needed in the future to validate this result.

## 5 Conclusion

Utilizing a comprehensive MR analysis, we delved into the causal relationships among plasma lipidome, circulating inflammatory proteins, and PD. Our findings revealed three negative and one positive causal effects between genetic predisposition to plasma lipidome and PD. Similarly, there were three negative and two positive causal effects between circulating inflammatory proteins and PD. Furthermore, we discovered that fibroblast growth factor 21 levels appear to mediate the pathway from phosphatidylcholine (14:0_18:2) levels to PD. Our research offers valuable insights into the intricate relationship between plasma lipidome and PD risk, which can not only guide future exploration of PD pathogenesis but also aid in the prediction, diagnosis, and even potential targeted therapy of PD.

## Data Availability

Publicly available datasets were analyzed in this study. This data can be found at: All data used in the study were obtained from published articles or publicly available GWAS platform (https://gwas.mrcieu.ac.uk/), and all data can be obtained for free.
