# Supplementary material for: Plasma lipidome, circulating inflammatory proteins, and Parkinson’s disease: a Mendelian randomization study
Source: Front Aging Neurosci. 2024 Sep 11;16:1424056. doi: 10.3389/fnagi.2024.1424056 (PMC11433008; doi:10.3389/fnagi.2024.1424056)
Supplement: Supplementary file 1 [file Data_Sheet_1.ZIP › Supplementary_Material/Supplementary_Material.docx]

Supplementary Material

# Supplementary Figures and Tables

## Supplementary Tables

**Supplementary Table 1.**3981 SNPs for the 179 plasma lipidome.

**Supplementary Table 2.** The causal effects of 179 plasma lipidome on PD.

**Supplementary Table 3：**Mendelian randomization pleiotropy residual sum and outlier (MR-PRESSO)

**Supplementary Table 4.** 1435 SNPs for the 91 circulating inflammatory proteins.

**Supplementary Table 5.** The causal effects of 91 circulating inflammatory proteins on PD.

**Supplementary Table 6.** Mendelian randomization Sensitivity analysis.

**Supplementary Table 7.** The causal effects of the reverse MR.

**Supplementary Table 8：**MR Steiger directivity test.

**Supplementary Table 9：**Causal effects of plasma lipidome associated with PD on circulating inflammatory proteins associated with PD.

## Supplementary Figures

**Supplementary Figure 1.** MR leave-one-out sensitivity analysis for plasma lipidome on PD.(A)Analysis for "Phosphatidylcholine (14:0_18:2) levels" on "PD"(B) Analysis for "Phosphatidylcholine (16:0_16:1) levels" on "PD"(C) Analysis for "Phosphatidylcholine (O-17:0_17:1) levels" on "PD"(D) Analysis for "Sphingomyelin (d38:1) levels" on "PD"

**Supplementary Figure 2.** Scatter plots for the effect of plasma lipidome on PD.(A)Analysis for "Phosphatidylcholine (14:0_18:2) levels" on "PD"(B) Analysis for "Phosphatidylcholine (16:0_16:1) levels" on "PD"(C) Analysis for "Phosphatidylcholine (O-17:0_17:1) levels" on "PD"(D) Analysis for "Sphingomyelin (d38:1) levels" on "PD"

**Supplementary Figure 3.**Forest plots for the effect of plasma lipidome on PD.(A)Analysis for "Phosphatidylcholine (14:0_18:2) levels" on "PD"(B) Analysis for "Phosphatidylcholine (16:0_16:1) levels" on "PD"(C) Analysis for "Phosphatidylcholine (O-17:0_17:1) levels" on "PD"(D) Analysis for "Sphingomyelin (d38:1) levels" on "PD"

**Supplementary Figure 4.**Funnel plots for the effect of plasma lipidome on PD.(A)Analysis for "Phosphatidylcholine (14:0_18:2) levels" on "PD"(B) Analysis for "Phosphatidylcholine (16:0_16:1) levels" on "PD"(C) Analysis for "Phosphatidylcholine (O-17:0_17:1) levels" on "PD"(D) Analysis for "Sphingomyelin (d38:1) levels" on "PD"

**Supplementary Figure 5.**MR leave-one-out sensitivity analysis for circulating cytokines on PD.(A) Analysis for "Fibroblast growth factor 21 levels" on "PD"(B) Analysis for "Interleukin-17A levels" on "PD"(C) Analysis for "Transforming growth factor-alpha levels" on "PD"(D) Analysis for "TNF-beta levels" on "PD"(E) Analysis for "Tumor necrosis factor receptor superfamily member 9 levels" on "PD"

**Supplementary Figure 6.**Scatter plots for the effect of circulating cytokines on PD.(A) Analysis for "Fibroblast growth factor 21 levels" on "PD"(B) Analysis for "Interleukin-17A levels" on "PD"(C) Analysis for "Transforming growth factor-alpha levels" on "PD"(D) Analysis for "TNF-beta levels" on "PD"(E) Analysis for "Tumor necrosis factor receptor superfamily member 9 levels" on "PD"

**Supplementary Figure 7.**Forest plots for the effect of circulating cytokines on PD.(A) Analysis for "Fibroblast growth factor 21 levels" on "PD"(B) Analysis for "Interleukin-17A levels" on "PD"(C) Analysis for "Transforming growth factor-alpha levels" on "PD"(D) Analysis for "TNF-beta levels" on "PD"(E) Analysis for "Tumor necrosis factor receptor superfamily member 9 levels" on "PD"

**Supplementary Figure 8.**Funnel plots for the effect of circulating cytokines on PD.(A) Analysis for "Fibroblast growth factor 21 levels" on "PD"(B) Analysis for "Interleukin-17A levels" on "PD"(C) Analysis for "Transforming growth factor-alpha levels" on "PD"(D) Analysis for "TNF-beta levels" on "PD"(E) Analysis for "Tumor necrosis factor receptor superfamily member 9 levels" on "PD"
